# Photodistributed Stevens–Johnson Syndrome/Toxic Epidermal Necrolysis

**DOI:** 10.1111/phpp.70053

**Published:** 2025-09-15

**Authors:** Sergio Castillo‐Pinto, Miguel Nieto, María Camila Marín Murillo, Adriana Cruz‐Garnica

**Affiliations:** ^1^ Pontificia Universidad Javeriana Bogotá Colombia; ^2^ Universidad Nacional de Colombia Bogotá Colombia; ^3^ Hospital Universitario de la Samaritana Bogotá Colombia

## 1 Introduction

Stevens–Johnson syndrome (SJS) and toxic epidermal necrolysis (TEN) are severe cutaneous adverse reactions, primarily drug‐induced, and histologically characterized by massive keratinocyte apoptosis mediated by cytotoxic T lymphocytes, leading to widespread epidermal necrosis [1]. Although the association with certain medications is well‐documented, various cofactors have been implicated in modulating the clinical expression and severity of the condition. Among these, exposure to ultraviolet (UV) radiation has been proposed as a potential trigger, although its pathogenic role remains not fully elucidated [2]. The photodistributed SJS/TEN phenomenon is rare and has been reported in a limited number of cases in the literature, associated with the intake of various drugs followed by sun exposure [3]. In this report, we present a 67‐year‐old patient with photodistributed SJS/TEN triggered by cephalexin, who initially received cyclosporine and methylprednisolone with a poor clinical response, requiring additional management with intravenous immunoglobulin.

## 2 Case Presentation

A 67‐year‐old man with a history of hypertension and hepatic neoplasm, who reported daily outdoor fieldwork with continuous exposure to UV radiation, presented with a 3‐day history of intense headache, fever, and conjunctival injection, followed by erosions on the vermilion, 48 h after initiating cephalexin. The condition progressed with the appearance of painful erythematous lesions and blisters on the extremities and trunk. Upon admission, the patient exhibited respiratory distress, hypoxemia, and tachycardia.

**FIGURE 1:**
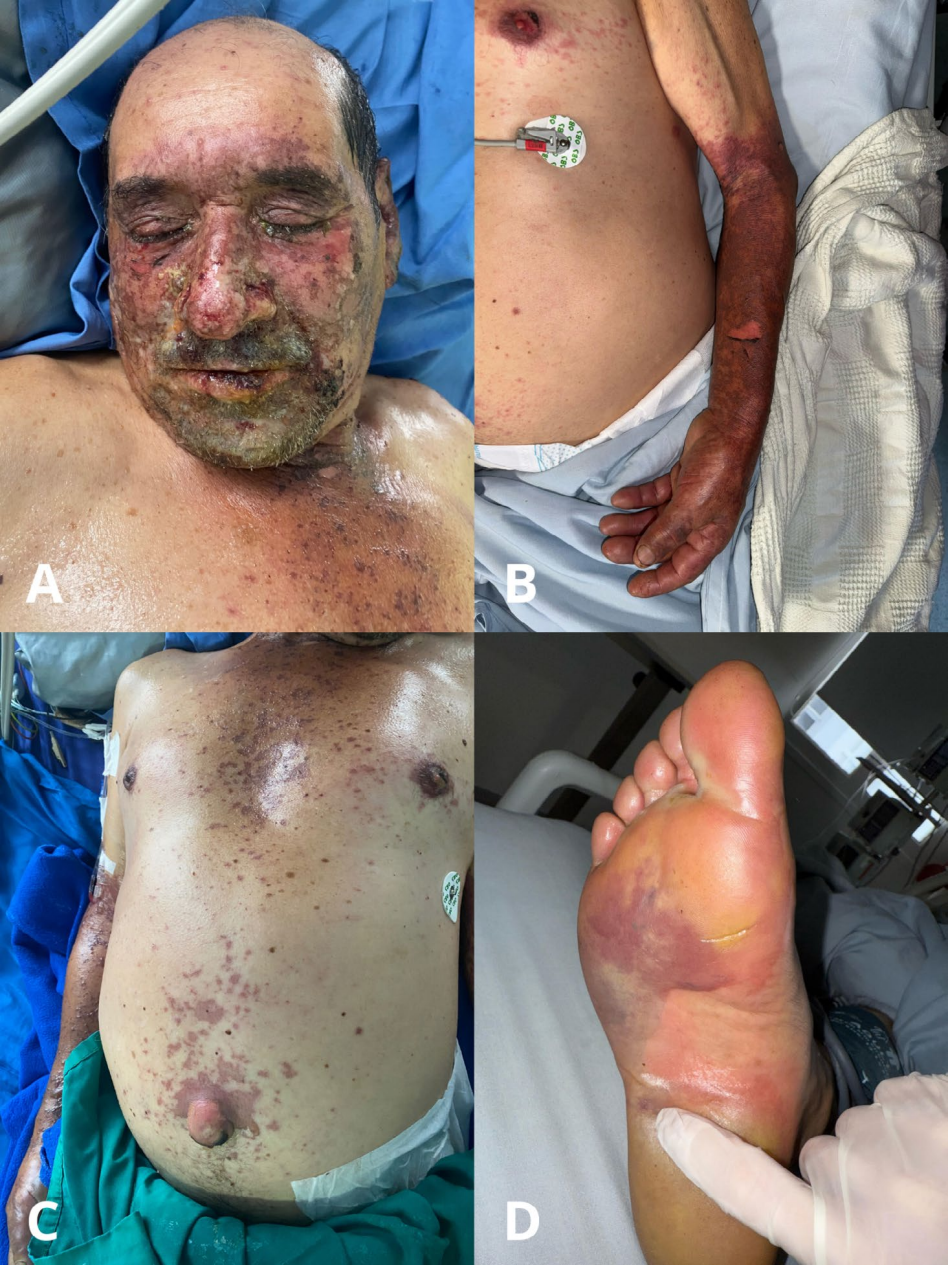
(A) Extensive erosions and honey‐colored crusts on the forehead, cheeks, eyelids, and vermilion border. (B, C) Erythematous–violaceous plaques with flaccid blisters on their surface, some erosions in photo‐exposed areas in trunk and arms. (D) Extensive blistering on the soles.

Physical examination revealed erythematous–violaceous plaques with flaccid blisters on their surface, with a positive Nikolsky sign, and some erosions, predominantly in photo‐exposed areas, including the central facial region, forearms, chest (V area), periumbilical skin, palms, and soles. Vermilion erosions with hemorrhagic crusts were also noted (Figure 1). The denuded area involved 20%–30% of the body surface area (BSA).

**FIGURE 2:**
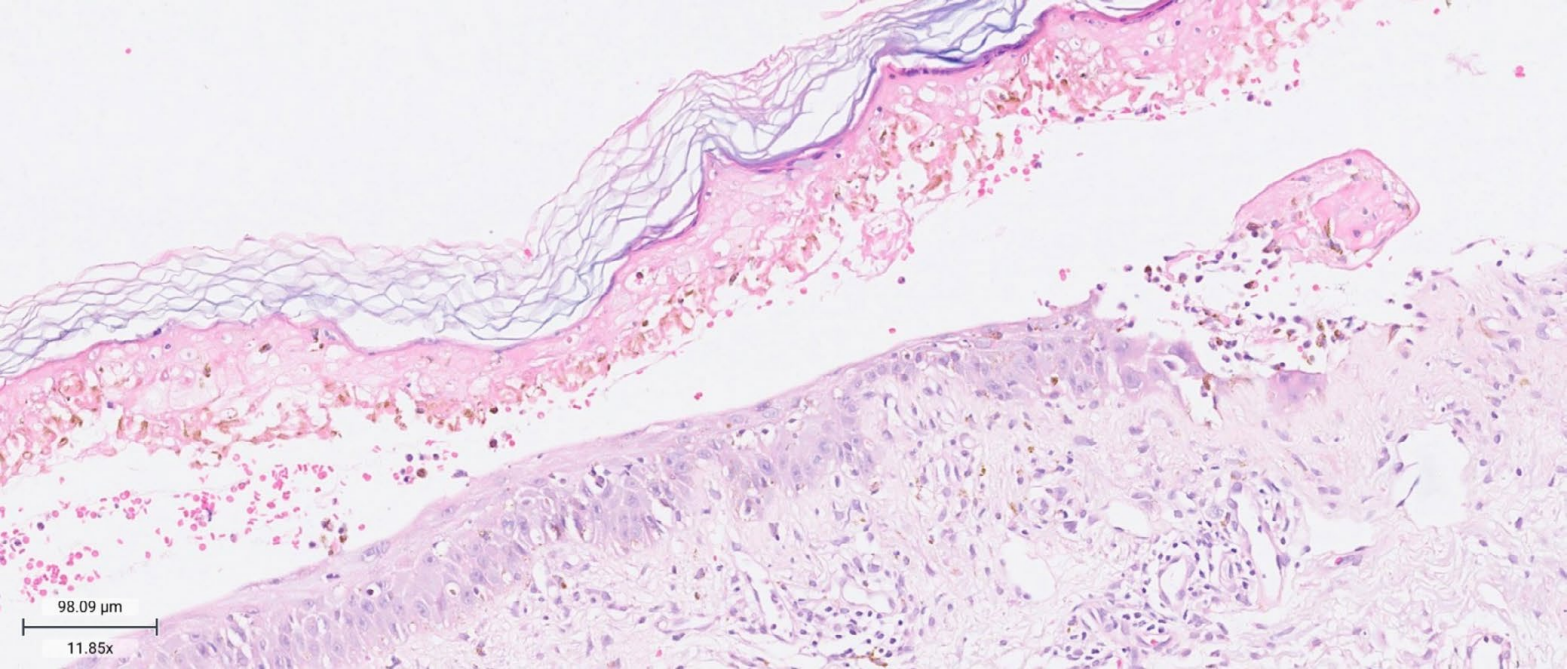
Histopathological findings (H&E × 40). Vacuolar interface damage in the epidermis, with numerous apoptotic bodies and lymphocyte exocytosis, associated with subepidermal cleft formation and full‐thickness epidermal necrosis, occasional eosinophils and neutrophils, with the presence of melanophages.

Acute lupus erythematosus TEN‐like, bullous lupus, and SJS/TEN were also considered as differential diagnoses. Skin biopsy was performed, reporting vacuolar interface damage in the epidermis, with numerous apoptotic bodies and lymphocyte exocytosis, associated with subepidermal cleft formation and full‐thickness epidermal necrosis, and in the dermis, occasional eosinophils and neutrophils, with the presence of melanophages. Laboratory workup showed negative antinuclear antibodies (ANAs), anti‐histone, anti–double‐stranded DNA, anti‐Smith, anti‐Ro, and anti‐La antibodies. Based on the histopathological findings in conjunction with the negative antibody panel, the diagnosis of SJS/TEN was established, with a SCORTEN score of 4 (Figure 2).

On the second day of hospitalization, treatment with cyclosporine at 3 mg/kg/day and pulses of methylprednisolone was initiated; after 8 days, no new blisters developed; however, clinical deterioration occurred, associated with secondary infection of periocular erosions, configuring sepsis. Blood cultures grew Gram‐positive cocci, for which vancomycin was administered.

Given the clinical decline related to infection and the SJS/TEN diagnosis, cyclosporin was discontinued, and intravenous immunoglobulin was initiated on Day 10, leading to improvement in skin lesions and general condition. Nevertheless, the patient died on Day 15 of hospitalization due to septic shock.

## 3 Discussion

SJS and TEN are rare, severe cutaneous reactions within the same disease spectrum, primarily differentiated by the extent of BSA involvement. While most cases are drug‐induced, a minority have been linked to *Mycoplasma pneumoniae* infection, vaccines, systemic diseases, or herbal medicines [1, 2].

UV radiation has been proposed as a cofactor in photodistributed SJS/TEN. The first case of photodistributed SJS, secondary to chloroquine, was reported in 1989 [4]. To date, only 14 cases have been reported in the literature, typically following drug ingestion and subsequent UV exposure within 24–36 h before symptom onset [3]. These cases often show faster onset (3–7 days), predominant involvement of photo‐exposed skin, though some lesions may appear in covered skin, and frequent mucosal lesions. Systemic symptoms such as headache, fever, and asthenia concomitant with skin or mucosal lesions have been reported in only one case [5]. This suggests that UV radiation may influence the clinical presentation and progression of the disease [3].

The underlying mechanism involves a type IV hypersensitivity reaction mediated by CD8^+^ T cells, with elevated granzymes, perforin, and Fas–FasL interactions [1, 2, 3]. UV radiation may act as a “second hit” by stimulating keratinocytes to release tumor necrosis factor and increase expression of intercellular adhesion molecule 1 (ICAM‐1) on antigen‐presenting cells, enhancing T‐cell activation in photo‐exposed areas. It may also upregulate FasL expression, promoting apoptosis [2, 3, 4, 5, 6]. Additionally, UV light may induce photoproducts and reactive oxygen species (ROS) that further contribute to keratinocyte damage [3].

Diagnosis relies on clinical findings, history of drug intake, and histopathology, typically revealing keratinocyte apoptosis, extensive confluent epidermal necrosis, vacuolar degeneration, and blister formation [7]. Management of SJS/TEN includes cyclosporine as first‐line therapy in patients with preserved renal function, showing benefits in reducing mortality, shortening hospital stay, and enhancing re‐epithelialization [7, 8]. In our case, it was selected as the initial treatment, showing a favorable early response. Other systemic therapies reported in the literature include corticosteroids, pentoxifylline, and antibiotics for secondary infections [1, 3]. IVIG is also considered an alternative treatment, supported by expert consensus. Although IVIG may contribute to improved survival and enhanced re‐epithelialization, it is associated with a wide range of adverse effects [7, 8, 9].

## Author Contributions

All authors take responsibility for the integrity of the data and the accuracy of the data analysis. All authors contributed to the manuscript conception, design, drafting, and revision of the article. All authors read and approved the final manuscript.

## Ethics Statement

All patients in this manuscript have given written informed consent for participation in the study and the use of their de‐identified, anonymized, aggregated data and their case details (including photographs) for publication. The project was reviewed and approved by the Ethics and Research Committee of the hospital, in accordance with applicable ethical guidelines.

## Conflicts of Interest

The authors declare no conflicts of interest.

## Data Availability

Data sharing not applicable to this article as no datasets were generated or analyzed during the current study.

## References

[1] O. A. Charlton , V. Harris , K. Phan , E. Mewton , C. Jackson , and A. Cooper , “Toxic Epidermal Necrolysis and Stevens‐Johnson Syndrome: A Comprehensive Review,” Advances in Wound Care 9, no. 7 (2020): 426–439. 32520664 10.1089/wound.2019.0977 PMC7307670

[2] K. Russomanno , A. DiLorenzo , J. Horeczko , et al., “Photodistributed Toxic Epidermal Necrolysis in Association With Lamotrigine and Tanning Bed Exposure,” JAAD Case Reports 14 (2021): 68–71. 34277913 10.1016/j.jdcr.2021.05.015 PMC8263525

[3] B. J. McKinley , M. E. Allen , and N. Michels , “Photodistributed Stevens‐Johnson Syndrome and Toxic Epidermal Necrolysis: A Systematic Review and Proposal for a New Diagnostic Classification,” European Journal of Medical Research 28, no. 1 (2023): 188. 37303053 10.1186/s40001-023-01142-2 PMC10259004

[4] B. Ordel , A. Sivayathorn , and H. Hönigsmann , “An Unusual Combination of Phototoxicity and Stevens‐Johnson Syndrome due to Antimalarial Therapy,” Dermatology 178, no. 1 (1989): 39–42. 10.1159/000248385 2917679

[5] R. Suarez Moro , L. Trapiella Martinez , E. Avanzas Gonzalez , J. Salas Puig , and C. Fernández Fernández , “Stevens‐Johnson Syndrome Secondary to Carbamazepine Mediated by Photosensitivity,” Anales de Medicina Interna 17 (2000): 105–106. 10829471

[6] L. J. Gaghan , M. M. Coates , L. N. Crouse , J. Miedema , J. E. Mervak , and C. M. Ziemer , “Photodistributed Toxic Epidermal Necrolysis,” JAMA Dermatology 158, no. 7 (2022): 787. 35507359 10.1001/jamadermatol.2022.1090 PMC9069340

[7] D. Creamer , S. A. Walsh , P. Dziewulski , et al., “UK Guidelines for the Management of Stevens‐Johnson Syndrome and Toxic Epidermal Necrolysis in Adults 2016,” Journal of Plastic, Reconstructive & Aesthetic Surgery 69, no. 6 (2016): e119–e153. 10.1016/j.bjps.2016.01.034 27287213

[8] H. C. Chang , T. J. Wang , M. H. Lin , and T. J. Chen , “A Review of the Systemic Treatment of Stevens–Johnson Syndrome and Toxic Epidermal Necrolysis,” Biomedicines 10, no. 9 (2022): 2105. 36140207 10.3390/biomedicines10092105 PMC9495335

[9] M. AlFada , H. Alotaibi , S. Alsharif , et al., “Systematic Review, Methodological Appraisal, and Recommendation Mapping of Clinical Practice Guidelines for Managing Patients With Stevens‐Johnson Syndrome and Toxic Epidermal Necrolysis,” Journal of Dermatological Treatment 36, no. 1 (2025): 2467751. 40010698 10.1080/09546634.2025.2467751

